# The invasive coral *Oculina patagonica* has not been recently introduced to the Mediterranean from the western Atlantic

**DOI:** 10.1186/s12862-015-0356-7

**Published:** 2015-05-05

**Authors:** Karine Posbic Leydet, Michael E Hellberg

**Affiliations:** Department of Biological Sciences, Louisiana State University, Baton Rouge, LA 70803 USA

**Keywords:** Introduction, Native invasive, Range expansion, *Oculina*, Mediterranean Sea

## Abstract

**Background:**

Effective policies, management, and scientific research programs depend on the correct identification of invasive species as being either native or introduced. However, many species continue to be misidentified. *Oculina patagonica*, first recorded in the Mediterranean Sea in 1966, is believed to have been introduced in anthropogenic times and expanding in a west to east direction. However, its present identification and status as a recently introduced species remain to be explored. In this study, we used multi-locus genetic data to test whether *O. patagonica* in the Mediterranean has been recently introduced from the western North Atlantic.

**Results:**

We found no genetic or historical demographic evidence to support a recent introduction of *O. patagonica* from the western North Atlantic or an expansion across the Mediterranean. Instead, Mediterranean and Atlantic populations are genetically distinct and appear to have begun diverging about 5 Mya. We also found evidence of a fossil record of *Oculina* spp. existing in the eastern North Atlantic millions of years before the present.

**Conclusions:**

Our results suggest that Mediterranean populations of *O. patagonica* have long been isolated from the western Atlantic, either in undetectable numbers or overlooked and undersampled sites and habitats, and have only recently been expanding to invasive levels as a result of environmental changes. Accurate identification of species’ invasive statuses will enable more effective research programs aimed at better understanding the mechanisms promoting the invasive nature of species, which can then lead to the implementation of efficient management plans.

**Electronic supplementary material:**

The online version of this article (doi:10.1186/s12862-015-0356-7) contains supplementary material, which is available to authorized users.

## Background

The number of introduced and invasive species is rising [1], rapidly altering ecosystems around the world, often by out-competing and displacing native species [2,3]. Beyond their direct impacts on native species, these invasive species can indirectly lead to cascading effects within a community [4], thereby threatening ecosystem functions. Studies concerning successful introduced and invasive species are key to exploring the mechanisms by which these species adapt to and alter their new environment. However, determining whether a species is native or introduced is first necessary, and this is not always an easy task.

**Table 1:** Analysis of molecular variance performed for all populations

**Source of variation**	**% variation**	**F-value**	**P-value**
Within Populations	58.9%	0.411	NA
Among Populations	8.4%	0.125	**<<0.01**
Among Groups	3.3%	0.328	**<<0.01**

Groups = western North Atlantic populations and Mediterranean populations. Significant P-values are in bold.

**Table 2:** **Analysis of molecular variance performed for Mediterranean populations only**

**Source of variation**	**% variation**	**F-value**	**P-value**
Within Populations	98.0%	0.02	NA
Among Populations	1.4%	0.014	0.256
Among Groups	0.6%	0.006	0.101

Groups = West (Spain and Italy) and East (Greece, Lebanon, and Israel).

**Table 3:** **Evidence ratios and ranks of all possible isolation with migration models**

**Model**	**k**	**log (P)**	**AIC**	**Δi**	**Model likelihoods**	**wi**	**Evidence ratio (best model)**
ABADE	4	−1.770	11.540	0.000	1.000	0.364	
ABC0D	4	−1.953	11.906	0.366	0.833	0.303	1.200
FULL	5	−1.446	12.891	1.351	0.509	0.185	1.965
ABBDE	4	−2.723	13.446	1.906	0.386	0.140	2.593
ABADD	3	−7.920	21.840	10.300	0.006	0.002	172.431
ABBDD	3	−8.351	22.702	11.163	0.004	0.001	265.416
ABCDD	4	−7.358	22.715	11.175	0.004	0.001	267.094
AAADE	3	−8.398	22.795	11.255	0.004	0.001	277.994
AACDE	4	−7.866	23.731	12.192	0.002	<10^−3^	443.989
AACDD	3	−10.835	27.671	16.131	<10^−3^	<10^−3^	3182.429
AAADD	2	−12.542	29.084	17.545	<10^−3^	<10^−3^	6452.997
ABCD0	4	−195.238	398.476	386.936	<10^−50^	<10^−50^	<10^50^
ABC00	3	−228.380	462.760	451.220	<10^−50^	<10^−50^	<10^50^
ABA00	2	−234.240	472.480	460.941	<10^−100^	<10^−100^	<10^100^
ABB00	2	−249.428	502.855	491.315	<10^−100^	<10^−100^	<10^100^
AAC00	2	−263.32	530.648	519.108	<10^−100^	<10^−100^	<10^100^
AAA00	1	−272.668	547.337	535.797	<10^−100^	<10^−100^	<10^100^

Evidence ratios and ranks calculated using model-based selection. For each model, the first three letters represent the three population parameters (*θ*_*1*_ *, θ*_*2*_, and ancestral *θ*), and the last two letters represent the two migration parameters (*m*_*1*_ and *m*_*2*_), in that order. The best model is the first model listed (ABADE) followed by the next best models in descending order.

**Table 4:** **Museum records of fossil specimens of** ***Oculina*** **spp. from the eastern Atlantic**

**Museum**	**Record number**	**Species**	**Location**	**Epoch or age**
Smithsonian National Museum of Natural History	USNM 64539	*Oculina* sp.	Indre-et-Loire, France	Eocene–Serravallian
	USNM I 80806	*Oculina crassoramosa*	France	Miocene
	USNM I 80807	*Oculina crassoramosa*	France	Miocene
	USNM I 80808	*Oculina solanderi*	Oise, France	Lutetian
	USNM I 80809	*Oculina raristella*	France	Eocene
	USNM I 80810	*Oculina* sp.	Oise, France	Lutetian
	USNM I 80811	*Oculina* sp.	Seine-et-Oise, France	Lower Lutetian
	USNM I 80812	*Oculina* sp.	Eure, France	Lutetian
Muséum National d’Histoire Naturelle	MNHN-F-M00169	*Oculina gemmata*	Calvados, France	Bathonian
	MNHN-F-M00170	*Oculina neustriaca*	Calvados, France	Bathonian
	MNHN-F-M00326	*Oculina crassoramosa*	Indre-et-Loire, France	Langhian
	MNHN-F-M00598	*Oculina crassoramosa*	Indre-et-Loire, France	Langhian
	MNHN-F-M00675	*Oculina raristella*	Oise, France	Lutetian
	MNHN-F-M00745	*Oculina crassoramosa*	Indre-et-Loire, France	Langhian
	MNHN-F-M00749	*Oculina crassoramosa*	Indre-et-Loire, France	Langhian
	MNHN-F-M01113	*Oculina explanata*	Sarthe, France	Cenomanian

**Figure 1:**
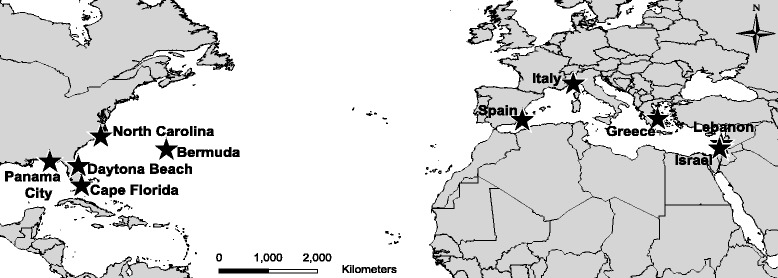
Map of collections sites of *Oculina* spp. populations used in this study. Populations along the western North Atlantic included North Carolina, three locations in Florida (Daytona Beach, Cape Florida, and Panama City), and Bermuda. Mediterranean populations included Spain, Italy, Greece, Lebanon, and Israel. This figure was created using maps freely available for use from ESRI.

**Figure 2:**
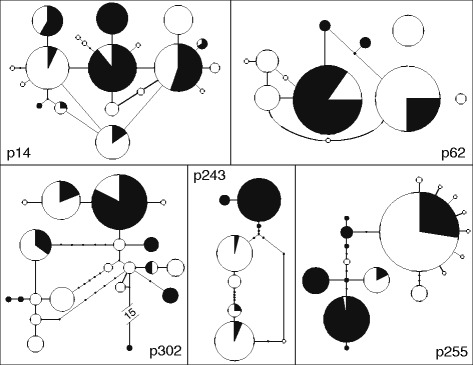
Haplotype networks of the five nuclear genes used in this study. Each pie graph represents an allele and the shades represent the proportion of individuals from the different populations that share that particular allele. White represents western North Atlantic *Oculina* spp. populations. Black represents Mediterranean populations of *Oculina patagonica*. Line segments connecting alleles represent a single mutation step separating the alleles, and small black dots represent inferred alleles not present in our dataset.

**Figure 3:**
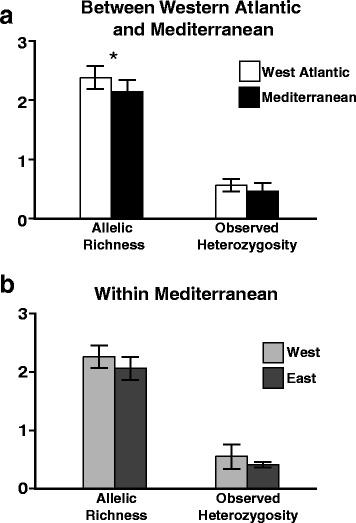
Comparison of allelic richness and observed heterozygosity. Means of allelic richness and observed heterozygosity compared between western North Atlantic and Mediterranean populations of *Oculina* spp. **(a)**, and between western Mediterranean (Spain and Italy) and eastern Mediterranean (Greece, Lebanon, and Israel) *Oculina patagonica* populations **(b)**. Bars represent standard deviations. Asterisks represent significant differences as determined by t-tests.

**Figure 4:**
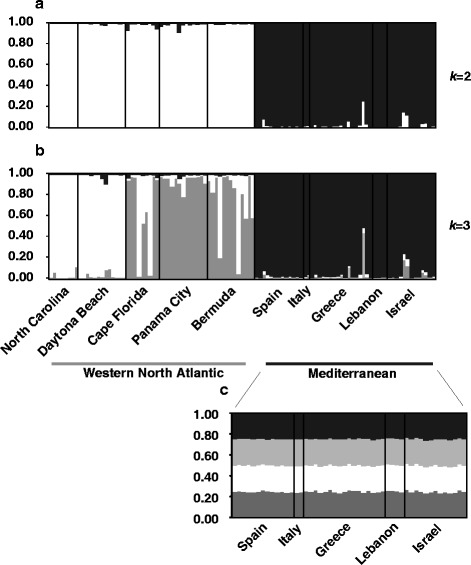
STRUCTURE bar plots. Each bar represents an individual. Individuals are grouped by populations along the x-axis. Along the y-axis is the probability of assignment to a particular population represented by different shades. When analyzing all populations, the Evanno method [43] determined that the mostly likely number of genetic clusters or populations (*k*) was 2 **(a)**. The visual representation of these two populations **(a)** shows that individuals cluster geographically (western North Atlantic versus Mediterranean), and that the Mediterranean populations are genetically distinct from the western North Atlantic populations. At *k* =3 **(b)**, the two main genetic clusters previously found in the western Atlantic [23] were recovered, while maintaining a genetically differentiated Mediterranean cluster. When analyzing the Mediterranean populations alone **(c)** there is no clear genetic structure across the Mediterranean, even though the Evanno method [43] determined that the most likely number of populations (*k*) was 4.

**Figure 5:**
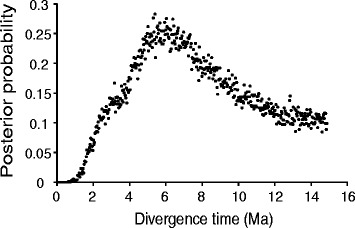
Posterior probability distribution for divergence times between western North Atlantic and Mediterranean populations of *Oculina* spp. Divergence time = 5.4 ± 2.0 million years ago.

**Figure 6:**
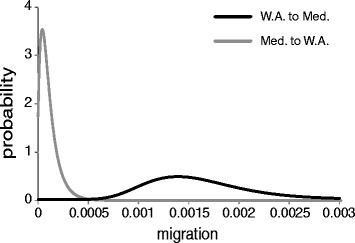
Posterior probability distributions for migration between western North Atlantic and Mediterranean populations of *Oculina* spp. Posterior probability distribution for migration (average number of migrants per 1000 generations) scaled by neutral mutation rate between western North Atlantic and Mediterranean populations of *Oculina* spp. Migration from the western North Atlantic to the Mediterranean was 0.00139 (90% highest posterior density interval = 0.0008–0.0.0024). Migration from the Mediterranean to the western North Atlantic was 0.000042 (0–0.002).

Introduced species can be mislabeled as native due to taxonomic misidentification [5,6]. Native species can also be misidentified as introduced. Zenetos et al. [7] reports that 23% of the 963 reportedly introduced species in the Mediterranean have been misidentified and therefore misclassified. Of the remaining 745 species, 13% remain questionable due to insufficient information and unresolved taxonomic status and many others maintain a “cryptogenic” status, as they cannot be reliably assigned to either “native” or “introduced” [8]. In other cases, species are mislabeled as introductions due to a lack of historical records of an obvious presence [7,9-11]. These misidentifications can have profound effects on the assessment of species status [12].

Invasive species are often assumed to have been introduced [9,10], however species can become invasive within their native range, usually due to human-mediated disturbances [13-15]. Effective policies, management, and scientific research programs depend on the correct identification of invasive species as being either native or introduced. Whereas the management of introduced invasives is more concerned with the introduction scenario and conditions enabling subsequent expansion, management of native invasives should ideally be more focused on the changes in environmental conditions that facilitate their invasive characteristics in their native habitat [11,16]. Therefore, to implement more effective research and management programs for invasive species, an invasive species must first be identified as either introduced or native.

An introduced invasive population can be distinguished from native invasive populations in several ways. An expanding introduced population is expected to be genetically similar to an external source population where it originated [17]. In contrast, a native invasive population will often be genetically distinct from populations outside its range [9,18]. Inferring the divergence time between populations from different ranges can also establish whether or not an invasive population coincides with a recent introduction (estimated divergence time will overlap with the present day), or whether it predates anthropological times (older divergence time) [19-21]. Finally, the presence of a fossil record for an invasive species or its progenitors can indicate that the species has a long presence in a particular region [10,11].

Corals of the genus *Oculina* were originally described from the southeastern coast of North America [22]. Although several nominal species exist in this region, Eytan et al. [23] found no genetic differences among shallow water populations of four named taxa (*O. arbuscula*, *O. diffusa*, *O. varicosa*, and, *O. robusta*), suggesting that these designations do not represent genetically distinct species. We will refer to these taxa collectively as “western Atlantic (WA) *Oculina* spp”.

An additional extant species, *Oculina patagonica*, occurs in the waters of the Mediterranean Sea [24,25]. *O. patagonica* was originally described from fossils from the southeastern coast of South America [24] (see Additional file 1: Figure S1), however reports of live specimens from South America are lacking and a recent survey of fouling communities in Argentinian ports failed to find any evidence of this species [26]. *O. patagonica* has been thought to have been introduced in anthropogenic times from the western south Atlantic to the western Mediterranean via shipping. *O. patagonica* was first reported from the harbor of Savona (Gulf of Genoa), Italy in 1966 [24] and soon after from the harbor of Alicante, Spain [27], 1000 km away. From the western Mediterranean, it is thought to have spread easterly, and while today reports of this coral in many locations reflect populations limited in number and range, populations in Spain, Greece, and Israel are well-established and expanding [24,25,27-38].

Alternatively to being recently introduced, *O. patagonica* may be a native species that has only recently been detected due to a recent expansion. The original description and identification of *O. patagonica* is based on fossil remains, not living counterparts [24]. This is problematic, as morphology is a poor delineation of coral species in general [39,40] and *Oculina* species in particular [23] (see Additional file 1: Figure S1). Given that no known populations of *O. patagonica* presently exist outside the Mediterranean, populations of WA *Oculina* spp. are currently the most likely source for a recent introduction.

Here, we ask whether *O. patagonica* has been recently introduced into the Mediterranean from the western North Atlantic, or whether it is an eastern Atlantic native only newly become invasive. First, we use multilocus genetic data to determine whether *O. patagonica* is genetically similar to or distinct from WA *Oculina* spp. If *O. patagonica* has been recently introduced from the western North Atlantic, we expect these populations to be genetically similar. Second, we estimate divergence time between *O. patagonica* populations and WA *Oculina* sp. populations and evaluate whether the estimate is consistent with an anthropogenic introduction. We also search museum collections for evidence of a fossil record of *Oculina* spp. in the eastern Atlantic, which would suggest that *Oculina* has a long history in this region. Finally, we explore whether patterns of genetic diversity in *O. patagonica* are consistent with a west to east expansion across the Mediterranean from a single point of introduction.

## Results

### Genetic diversity and population subdivision

We genotyped 122 samples of *Oculina* spp. from the western North Atlantic (n = 56) and Mediterranean (n = 66) for the mitochondrial *COI* gene and five nuclear genes. Western North Atlantic populations included North Carolina, Daytona Beach, FL, Cape Florida, FL, Panama City, FL, and Bermuda. Mediterranean populations of *O. patagonica* included Spain, Italy, Greece, Lebanon, and Israel (Figure 1). A total of 17 individuals from Spain, Greece, and Israel were removed from the dataset because they shared a multilocus genotype with another individual in the same population, so the final nuclear data set contained 105 individuals (see Additional file 2: Table S1). All *O. patagonica* samples shared the same *COI* haplotype common to 98% of WA *Oculina* spp. (see Additional file 3: Figure S2). Because *COI* was nearly invariant, as expected due to the conservation of anthozoan mitochondrial DNA [41], we used only the five nuclear genes in all analyses. GARD did not detect recombination within any of these five gene regions.

Haplotype networks revealed that, while populations in the western North Atlantic and the Mediterranean share many alleles at all five loci, all markers possess several private alleles unique to just one region (Figure 2). Specifically, for three genes (p14, p62, and p302), over half of the total alleles for each of those genes are unique to the Atlantic. In contrast, the Mediterranean harbors fewer private alleles for all genes (Figure 2). T-tests revealed that allelic richness was greater in the western North Atlantic than in the Mediterranean (Figure 3a), although this difference is not large (western North Atlantic mean = 2.38 ± 0.09; Mediterranean mean = 2.14 ± 0.09), and therefore likely not biologically significant. Observed heterozygosity did not differ between the western Atlantic and Mediterranean (Figure 3a), nor did allelic richness or observed heterozygosity between western Mediterranean (Spain and Italy) and eastern Mediterranean (Greece, Lebanon, and Israel) populations (Figure 3b).

AMOVA revealed significant subdivision among all populations, as well as between western North Atlantic and Mediterranean populations (Table 1). However, AMOVA conducted on Mediterranean populations alone revealed no significant subdivision, either among populations or between the west and east (Table 2). Instead, variation within populations accounted for 98% of observed variation. These findings suggest that while the western North Atlantic populations are genetically distinct from those in the Mediterranean, populations within the Mediterranean are genetically similar to each other.

To further test for more subtle genetic differentiation, we used STRUCTURE 2.3.4 [42] and the Evanno method [43] implemented in STRUCTURE HARVESTOR [44] to detect differentiated populations (*k*). When all populations were analyzed according to the Evanno method [43], the most likely *k* was two. The visual representation of these two genetic clusters (Figure 4a) shows that the western North Atlantic, including Bermuda, and Mediterranean form distinct genetic clusters. At *k* =3, STRUCTURE recovered the two main genetic clusters previously found in the western North Atlantic [23] in addition to the Mediterranean cluster (Figure 4b). When analyzing the Mediterranean populations alone, the Evanno method [43] determined that the mostly likely *k* =4, although the Δ*k*’s for the range of *k* tested were very low and similar, suggesting a lack of biologically meaningful clusters. Indeed, the visual representation fails to show any clear individual assignments and geographic association of these clusters, consistent with the Mediterranean populations being genetically similar across the region (Figure 4c). STRUCTURAMA runs corroborated these STRUCTURE results.

### Divergence time

We estimated the time of divergence between western North Atlantic and Mediterranean populations using IMa [45]. We found that the populations diverged 5.4 ± 2.0 million years ago, long before recent times (Figure 5). The best supported IM model (Table 3) had two parameters for population size and two for migration, suggesting that migration has played a role in the history of WA *Oculina* spp. populations and *O. patagonica* in the Mediterranean. Models of strict isolation were thousands of times less likely than the best model. Migration from the western North Atlantic to the Mediterranean was greater (0.00139 [90% highest posterior density interval =0.0008–0.0024]) than the reverse migration (0.000042 [0–0.002]) (Figure 6). In fact, the next best model was one in which migration from the Mediterranean to the western North Atlantic was equal to 0.

### The fossil record

We explored online databases of museum collections for a fossil record of *Oculina* spp. in the eastern Atlantic and/or the Mediterranean. We found 16 fossil specimens of *Oculina* spp. in two independent museum collections: the Smithsonian National Museum of Natural History’s Department of Invertebrate Zoology (see Additional file 1: Figure S1), and the Muséum National d’Histoire Naturelle Paléontologie. All specimens originated from north-northwestern France. The estimated ages of the specimens range broadly, with most from the Eocene (56–34 Ma) to the Miocene (23.03–5.332 Ma) (Table 4).

### Population expansion within the Mediterranean

To test for a past population expansion within the Mediterranean Sea, we used LAMARC 2.0 [46]. The overall population growth rate across all genes and replicates was −65, indicating that the population has not been expanding. We treated the Mediterranean as a single population, since we did not detect significantly differentiated populations within the Mediterranean (Figure 4c). We also performed analyses on the three Mediterranean populations with the largest sampling sizes (Spain, Greece, and Israel) separately, and found similar results.

## Discussion

### *Oculina patagonica* has not been recently introduced into the Mediterranean

Our data show that Mediterranean populations of *O. patagonica* are genetically distinct from WA *Oculina* spp. populations (Figure 4a). While *Oculina* spp. populations from either side of the Atlantic share many alleles for all markers, there were notable private alleles for both regions (Figure 2). Contrary to expectations for a recently introduced and expanding species, Mediterranean *O. patagonica* harbors genetic diversity on par with long-established WA *Oculina* spp. populations (Figure 3a). Our IMa results reveal that *O. patagonica* and WA *Oculina* spp. populations diverged millions of years ago (Figure 5). Taken together, our results suggest that while *O. patagonica* populations from the Mediterranean are closely related to WA *Oculina* spp. populations, they are genetically differentiated from them and have not been introduced into the Mediterranean from the western North Atlantic in anthropogenic times. Although we did not include *Oculina* spp. samples from the Caribbean, Bermuda likely represents the Caribbean given that in other broadcast spawning corals little genetic variation has been found between the Caribbean and the western Atlantic [47].

While there are many similar examples of misidentified native species [9,11,19], and see below, contrary to our findings for *O. patagonica*, many species have been introduced into the Mediterranean. A well-known example is the green alga, *Caulerpa taxifolia* [48]. Using nuclear sequence data, Jousson et al. [49] determined that this species was introduced into the Mediterranean from an aquarium in Monaco, which maintained an algal strain of unknown geographical origin cultivated in western European aquaria. Mitochondrial sequence data was used to determine that a Mediterranean clade of sea squirts, *Clavelina lepadiformis*, was recently introduced from eastern Atlantic populations [5]. The Mediterranean Sea has experienced an influx of introduced species in recent decades [50], which has been attributed to increased sea temperatures, along with coincident range expansions of introduced species and range shifts of native ones [51].

### Where did *O. patagonica* originate?

The original hypothesis for the origin of Mediterranean *O. patagonica* suggested that, based on its identification, it must have been introduced from South America, where the only evidence (fossils) of this species existing outside the Mediterranean resides [24]. However, reports of live specimens of *O. patagonica* in South America are lacking, and a recent survey of fouling communities in Argentinian ports failed to find any evidence of this species [26]. If living *O. patagonica* are not present in the western South Atlantic, they could not have been recently introduced into the Mediterranean from this region. Although it is possible that *O. patagonica* still resides in the western South Atlantic in low undetected numbers or habitats, there are no grounds to suggest *O. patagonica* recently originated from South America until (if) those specimens are found.

If *O. patagonica* has not recently travelled east across the Atlantic to the Mediterranean, then where did it originate? We found records for 16 fossil specimens of *Oculina* spp. from France (Table 4). Along with these multiple records from two museums, *Oculina* spp. fossils have also been reported from the Danish Basin during the Middle Danian (about 64 Mya) [52] and south Aquitaine, France, during the Late Oligocene (about 25 Mya) [53]. Although all of these fossils originated from outside of the Mediterranean, they suggest that the genus *Oculina* has long been present in the eastern North Atlantic.

A long presence in the eastern Atlantic is consistent with our genetic data, which suggest that the western North Atlantic and Mediterranean populations diverged 5.4 ± 2.0 million years ago (Figure 5). This coincides with the Late Miocene Messinian Salinity Crisis (5.33 Ma), when sea levels in the Mediterranean basin dropped, separating it from the Atlantic and killing off many marine species [54,55]. The asymmetric migration in the history of *Oculina* spp. (Figure 6), with a greater inferred migration from the western North Atlantic to the Mediterranean, may reflect the repopulation of the Mediterranean with Atlantic aquatic fauna following the Messinian Salinity Crisis [54-56]. However, an ancient introduction would likely leave behind a fossil record, and we found no *Oculina* spp. fossils from within the Mediterranean. This may be due to undiscovered or undocumented fossils, but could also indicate that *O. patagonica* was more recently introduced from elsewhere, likely the eastern North Atlantic [57]. Our finding of no genetic structure within the Mediterranean also suggests that it may not have an ancient presence there. Further survey efforts are needed to determine whether extant *Oculina* spp. populations exist in the eastern North Atlantic, and whether they are the source of *O. patagonica*.

Another hypothesis for the origin of *O. patagonica* lies along the western coast of Africa. *Schizoculina africana* has both a fossil and living presence in Cape Verde [58,59]. Originally known as *Oculina africana*, this species was split to form a new genus (*Schizoculina*) due largely to a unique way in which polyps bud [24,60]. However, dual modes of budding have been reported within a single coral species [61] and may therefore not be a good diagnostic trait to differentiate species. Future genetic work is needed to investigate whether *Schizoculina africana* and *Oculina patagonica* are in fact conspecific, and whether *O. patagonica* originated from the northwestern coast of Africa.

Oculinidae is a taxonomically confused family [39,62] in need of a more in depth genetic study to better understand the relationships between and within the genera and species in this family. As indicated by mitochondrial and nuclear genes, Oculinidae is paraphyletic, and *Oculina* is more closely related to some members of different families (Faviidae, Caryophylliidae, and Rhizangiidae) than to some members of its own family. Thus, representatives from some extra-familial genera with which *Oculina* has sometimes been allied (*Astrangia* of the Rhizangiidae, *Cladocora* of the Caryophylliidae) [24] should also be included in future efforts to trace the origins and taxonomic classification of *Oculina patagonica*.

### The geographical expansion of *O. patagonica* in the Mediterranean

Direct observations testify to *O. patagonica’s* increase in abundance at shallow depths at many localities in the Mediterranean over the past 20 years [32,34]. Along the Catalan coast, the species spread from just one location in 1992 to 43 by 2010, a rate of northward expansion of 22 km per year [32]. In 2005, a few colonies of *O. patagonica* were first reported from a single site in the Saronikos Gulf of the Aegean Sea [29]. By 2009, *O. patagonica* could be found in 45 of 54 surveyed sites in this region [34].

Fine et al. [25] proposed that *O. patagonica* has been expanding west to east, just as first reports of its presence have. Our tests, however, did not detect a genetic signal of expansion across the Mediterranean. While this may result from low power, the proposed west to east spread is also opposite to most other range expansions in the Mediterranean, which have occurred in a north-westward direction in response to increasing sea temperature [51]. Because in the Mediterranean temperature increases from east to northwest, and rising temperatures have been proposed to be promoting the range expansion of *O. patagonica* [32], a west to east expansion would be contrary to expectation, unless it was introduced into the western Mediterranean Sea, which our tests failed to support. Furthermore, some recent first reports have come from the western Mediterranean [30], and *O. patagonica* was first reported from the Levant prior to Greece [28,29,35]. Finally, if *O. patagonica* was first established in the western Mediterranean and only more recently in the east, then the western populations would likely harbor more genetic diversity; however, we found similar levels of genetic diversity across the Mediterranean. Alternatively, *O. patagonica* could be moving into the Mediterranean from elsewhere in the eastern North Atlantic, but in sufficient numbers to not leave a genetic signature of expansion.

Despite lack of evidence for a demographic expansion from west to east, it appears that *O. patagonica*’s invasive behavior may have “expanded” west to east. Serrano et al. [32] report an expansion along the Spanish coast from 1992–2010. Salomidi et al. [34] reported a later spread along the coast of Greece from 2005–2009. While this eastward trend may owe to chance, it could also be due to human-mediated modifications of shallow coastal habitats occurring earlier in the west, or limiting conditions in the east [25] that populations have adapted or acclimated to overtime [63].

### *O. patagonica* is native species recently turned invasive

It seems most likely that *O. patagonica* has always existed somewhere in the eastern Atlantic and has recently become invasive in the Mediterranean, expanding in local regions in response to environmental change [51], likely mediated by human-modifications of coastal habitats [32,34]. In a similar way, the snowflake octocoral, identified in Hawai'i as *Carijoa riisei*, was believed to have been recently introduced from its native range in the Caribbean. However, Concepcion et al. [9] used mitochondrial and nuclear sequence data to compare the Hawaiian populations to worldwide populations of *Carijoa* and found that the Hawaiian populations were not genetically similar to the Caribbean and therefore did not originate from there. The originally misidentified native diatom *Didymosphenia geminata* remained undetected in its native range for decades before blooms were documented in the 1990s [64]. Today, this native invasive alga is rapidly expanding locally in response to environmental changes [11,64]. The gastropod, *Littorina littorea*, has long been thought to have been recently introduced to North American from Europe. However, both mitochondrial and nuclear sequence data indicated that the North American and European populations diverged thousands of years ago. This native gastropod is also believed to have begun expanding along the coast of New England as a result of environmental changes [19].

Identifying additional mechanisms that facilitate invasiveness in *O. patago*nica will require work aimed at better identifying and characterizing the source populations and population dynamics of well documented locally expanding *O. patagoncia* populations within the Mediterranean, such as along the coasts of Spain [32] and Greece [34]. The conditions at these invasion localities can then be compared to conditions where *O. patagonica* exits but is not to date invasive to better understand the mechanisms driving its expansion. Such studies may also aid in assessing the future of the newly discovered coral species, *Oulastrea crispate*, in the Mediterranean as it too is expected to rapidly expand its range [65].

Additional studies are also needed to better understand the ecological consequences of the expansion of *Oculina patagonica*. While marine range shifts may occur at a slower rate than marine introductions, their potential effects on the community are likely to be just as significant [66]. *O. patagonica* has been shown to successfully compete with bryozoan *Watersipora* sp. [67]. Serrano et al. [68] have reported a shift from macroalgal to *O. patagonica* dominance in the Mediterranean. Given that macroalgae are important primary producers, this shift may result in significant changes in ecosystem functioning.

Corals are currently facing worldwide declines as a result of stresses, including increasing sea temperatures, disease, and other anthropogenic disturbances [69-71]. Understanding the factors and characteristics that promote resilience in *O. patagonica* in the midst of environmental change may shed light into assessing and managing the long-term success of corals that are currently at risk.

## Conclusions

Despite years of maintaining that *Oculina patagonica* is a recently introduced coral species in the Mediterranean, we found no genetic or historical demographic evidence to support that claim. Our results suggest that Mediterranean populations of *O. patagonica* have long been isolated from WA *Oculina* spp., and have only recently become invasive in the Mediterranean, most likely due to environmental changes. We advise against hastily identifying a previously unknown species as being introduced without detailed genetic analyses and comparisons to potential source populations. Accurate identification of species’ invasive statuses will enable more effective research programs aimed at better understanding the mechanisms promoting the invasive nature of species, which can then lead to the implementation of efficient management plans.

## Methods

### Sampling and genotyping

Mediterranean samples (n = 66) of *Oculina patagonica* were collected from Spain, Italy, Greece, Lebanon, and Israel during the summers of 2011–13 (see Additional file 2: Table S1; Figure 1). Individual colonies were sampled by chipping off a small piece of skeleton containing coral tissue and preserving it in 95% ethanol. Samples were generally 10 m from conspecifics and not physically connected to them to avoid collecting clonemates.

Western North Atlantic *Oculina* spp. samples (n = 56) consisted of a subset of populations along the coast of the eastern United States from Eytan et al. [23]. The four populations (North Carolina, Daytona Beach, Cape Florida, Panama City) were chosen to represent the two geographic genetic clusters (North Carolina and Daytona Beach = northern cluster; Cape Florida and Panama City = southern cluster) and include three nominal species: *O. arbuscula*, *O. varicosa*, and *O. diffusa,* although Eytan et al. [23] found no genetic differences among these named taxa. We also obtained 13 new samples of nominal species *O. diffusa* and *O. varicosa* from Bermuda. We will refer to these samples collectively as “Western Atlantic (WA) *Oculina* spp”. All sampling was conducted by or with local collaborators in accordance with local and CITES regulations.

We extracted genomic DNA using QIAGEN DNeasy Kit following the manufacturer's protocols with the following modifications. We lysed tissues at 56°C overnight. We added 200 μl elution buffer and incubated at room temperature for an hour prior to the final centrifuge step. All individuals were genotyped, either previously or in this study, for the mitochondrial cytochrome oxidase I (*COI*) gene and the coding region of five nuclear genes (see Additional file 4: Table S2). *COI* was genotyped using previously deigned primers [72]. Three of the nuclear genes (putatively: *fatty acid elongase, elongation factor 1α, and tachylectin-2 motif*) were previously developed to assess subdivision in WA *Oculina* spp. populations [23], so only the 13 Bermuda and 66 Mediterranean samples were genotyped for these markers here. Two new nuclear markers (putatively: *crystalline and S-adenosylmethionine synthetase*) were developed using an expressed sequence tag (EST) library [23]; all samples were genotyped for these.

Polymerase chain reaction (PCR) amplifications were conducted in 25 μl reactions consisting of 2.5 μl of 10x buffer, 10 μM of dNTPs and each primer, and 0.2 μl of One TaqTM DNA polymerase (New England Biolabs Inc.). Amplifications were performed in a Bio-Rad T100 thermocyler under the conditions outlined by Eytan et al. [23]. Samples were sequenced in both directions using BigDye v3.1 on an ABI 3130XL at the Louisiana State University Genomics Facility. Sequences were aligned and edited using Geneious 4.5.5 [73]. Sequences obtained from Eytan et al. [23] were trimmed to align to sequences generated in this study. To resolve alleles in heterozygous individuals, we employed a Bayesian statistical method implemented in PHASE 2.1 [74-76]. Individuals with alleles that could not be phased to a probability >90% were cloned using the Invitrogen TOPO TA kit following the manufacture's protocols. At least eight clones per reaction were sequenced to identify the two alleles present in a sample. The phased individuals derived from the cloning reactions were then added to the ‘known’ PHASE file and the data sets were re-analyzed. This process was repeated until the phase of all individual genotypes was recovered with >90% probability. Individuals heterozygous for an insertion/deletion were resolved using CHAMPURU 1.0 [77]. In the end, we were able to successfully resolve all 122 individuals’ multilocus genotypes.

To prevent clonal reproduction from skewing subdivision and genetic diversity measures, we removed individuals that shared a multilocus genotype with another individual in the same population. The final nuclear data set contained 105 individuals (see Additional file 2: Table S1). Measures of genetic diversity for each nuclear marker were calculated in DnaSP [78,79] (see Additional file 4: Table S2). We tested each gene region for intralocus recombination using GARD implemented in Hy-Phy [80-82].

### Genetic diversity and population subdivision

To visualize the relationships among alleles, we constructed haplotype networks for each locus using statistical parsimony implemented in TCS 1.21 [83]. These networks reveal how alleles for a particular gene are shared among individuals from different populations. We calculated allelic richness for all populations using FSTAT 2.9.3.2 [84] and calculated observed heterozygosity using GENODIVE [85]. To test whether Mediterranean populations harbor less genetic diversity than western Atlantic populations, we compared their average allelic richness and observed heterozygosities using two-sample one-tailed t-tests in GraphPad Prism 5. We performed similar comparisons between western Mediterranean (Spain and Italy) and eastern Mediterranean (Greece, Lebanon, and Israel) populations using two-sample two-tailed t-tests. To test for hierarchical genetic subdivision, we performed Analyses of Molecular Variance (AMOVA) implemented in GENODIVE [85] for all populations combined, and for only Mediterranean populations.

The Bayesian clustering analysis implemented in STRUCTURE [42] has been used often to infer species introductions and identify potential source populations [17]. Here, we used STRUCTURE 2.3.4 [42] to test whether *O. patagonica* populations in the Mediterranean are genetically similar to or distinct from WA *Oculina* spp. populations. We first analyzed all populations together, and then analyzed the pool of Mediterranean populations separately. We ran the program for 1 million MCMC steps and discarded the first 100,000 steps as burn-in. We used the more conservative admixture model with uncorrelated allele frequencies. We performed 10 iterations for each inferred number of genetic clusters, *k*. We used the Evanno method [43] implemented in STRUCTURE HARVESTOR [44] to determine the most likely number of genetic clusters, *k*, in the data. We also used STRUCTRAMA 2.0 [86] to explicitly estimate *k* without *a prior* assignment of a range of *k*, as in STRUCTURE. Each trial was run for 20 million generations, sampling every 100, discarding the first million as burn-in. We ran four chains at a temperature of 0.2, and we employed a variety of model options.

### Divergence time

We estimated the time of divergence between the western North Atlantic and Mediterranean populations using a coalescent-based method implement in IMa [45]. IMa uses Markov Chain Monte Carlo (MCMC) simulations of gene genealogies to estimate the divergence time (*t*), genetic diversities (*θ*_*1*_*, θ*_*2*_, and ancestral *θ*) and migration rates (*m*_*1*_ and *m*_*2*_) for two populations. To convert divergence time (*t*), which is scaled by mutation in IMa, to years, we used the average nuclear substitution rate for *Porites* corals of 0.138% per Ma [87], since a rate for *Oculina* corals is unavailable, which was converted to a rate per locus per year for each marker (see Additional file 4: Table S2). Given that the sequence alignments showed sites with more than two variants and/or haplotype networks contained multiple reticulations, we used the finite-sites model for all genes.

We first performed several IMa runs, subsequently adjusting the upper bounds on the parameter priors, to determine the most efficient search parameters. We then ran four runs that differed only in the starting seed for 3,000,000 total steps sampling every 100 steps for a total of 30,000 saved genealogies following a burn-in of 500,000 steps. The runs yielded similar results. We therefore combined the runs and estimated all parameters and performed nested model testing on the total saved genealogies in IMa’s L mode (load trees). IMa analyses were conducted with high performance computational resources provided by Louisiana State University [88].

We recorded the maximum-likelihood estimate from the posterior probability distribution for divergence time, adjusted with a two year generation time [25] and its credibility interval based on the shortest parameter interval containing 90% of the area under the posterior distribution curve. Because the upper end of the posterior probability distribution did not drop to zero (Figure 5), we used the lower bound on the distribution as the parameter value at which the probability dropped to zero at the upper bound [89]. To evaluate all possible scenarios of divergence, which differ in the number of unique divergence parameters and therefore divergence complexity, we used model-based inference and model-based selection to calculate evidence ratios and rank all possible models [90-92].

### Fossil record search

We searched museum collections for evidence of a fossil record of *Oculina* spp. in the eastern Atlantic and/or the Mediterranean. First, we explored the Smithsonian National Museum of Natural History’s Department of Invertebrate Zoology records by performing a keyword search of *Oculina* on the IZ collections database website [93]. We also explored the Muséum National d’Histoire Naturelle Paléontologie collections database by performing a general search for *Oculina* on the collections website [94]. From both lists of matches, we searched for fossil specimens of *Oculina* spp. from eastern Atlantic and Mediterranean countries and recorded the catalog number, species name, location, and geologic age. Several of the specimens from the Smithsonian National Museum of Natural History were observed during a visit to the museum in January 2013.

### Population expansion within the Mediterranean

To test for expansion in the Mediterranean, we used LAMARC 2.0 [46], which estimates parameters including population growth rate using coalescent theory and Metropolis Monte Carlo sampling technique. Three replicates were each run using a Bayesian search strategy and a single final chain. Following a burn-in of 500,000, 5 million trees were sampled every 100 step. Three simultaneous searches were performed at heating temperatures of 1, 1.2, and 1.3, and a swap interval of 10. Trial runs were first conducted and the output files examined in the program TRACER 1.5 [95] to adjust the parameter bounds and assess the run. A “good run” was one in which both the effective sample size (ESS) values were great than 200 and trace plots for each parameter were stationary. LAMARC calculates the overall growth rate across all genes and replicates. Positive values of growth rate indicate that the population has been growing, while negative values indicate that it has been shrinking.

## Availability of supporting data

Haplotypes for *COI*, p14, p62, and p302 for western North Atlantic *Oculina* spp. populations obtained by Eytan et al. [23] are available on GenBank [FJ966395–FJ966875]. Haplotypes generated here are available on The European Nucleotide Archive [LN613417–LN614380].

## Additional files

Additional file 1: Figure S1. COI Neighbor-Joining Tree. Neighbor-joining tree constructed using *COI* haplotypes from western North Atlantic *Oculina* spp. populations and *O. patagonica* populations from the Mediterranean, with *Solenastrea hyades* as the outgroup. Numbers represent the number of individuals from each locality that share that haplotype. The tree shows that *O. patagonica* (bolded) shares the same haplotype common to most western North Atlantic *Oculina* spp. Additional file 2: Table S1. Collection sites of all *Oculina* spp. samples used in this study. Additional file 3: Figure S2. Photographs of *Oculina* spp. specimens. A–D are *Oculina* spp. fossil specimens from the Smithsonian National Museum of Natural History. A and B are *O. patagonica* from South America (USNM 75199 and USNM 75205, respectively). C and D are *O. crassoramosa* from France (USNM I 80807). E is a skeletal specimen of extant *O. patagonica* from the eastern Mediterranean. F is a skeletal specimen of extant *O. diffusa* from Panama City, Florida (USA). Additional file 4: Table S2. Nuclear markers used to genotype all *Oculina* spp. samples in this study.
