# Supplementary figures and images for: The invasive coral Oculina patagonica has not been recently introduced to the Mediterranean from the western Atlantic
Source: BMC Evol Biol. 2015 May 5;15:79. doi: 10.1186/s12862-015-0356-7 (PMC4418043; doi:10.1186/s12862-015-0356-7)

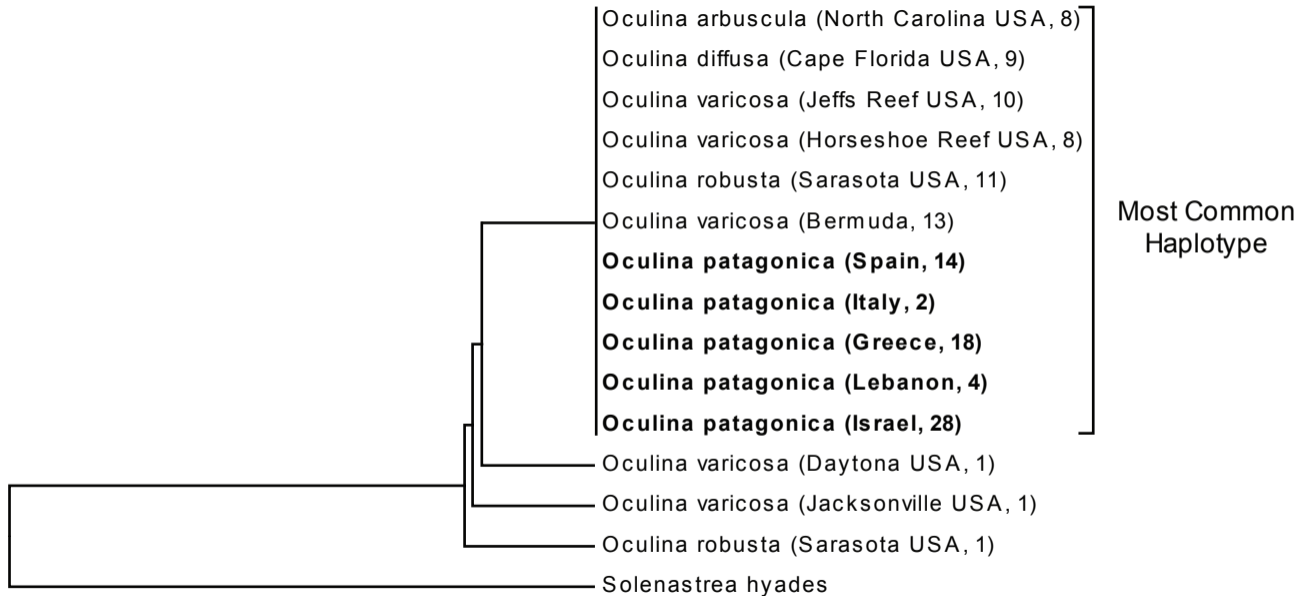

Supplement: Additional file 1: Figure S1. — COI Neighbor-Joining Tree. Neighbor-joining tree constructed using COI haplotypes from western North Atlantic Oculina spp. populations and O. patagonica populations from the Mediterranean, with Solenastrea hyades as the outgroup. Numbers represent the number of individuals from each locality that share that haplotype. The tree shows that O. patagonica (bolded) shares the same haplotype common to most western North Atlantic Oculina spp. [file 12862_2015_356_MOESM1_ESM.pdf]

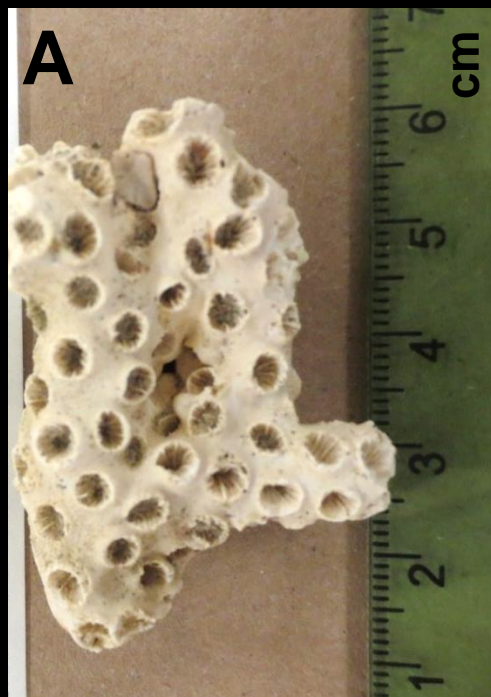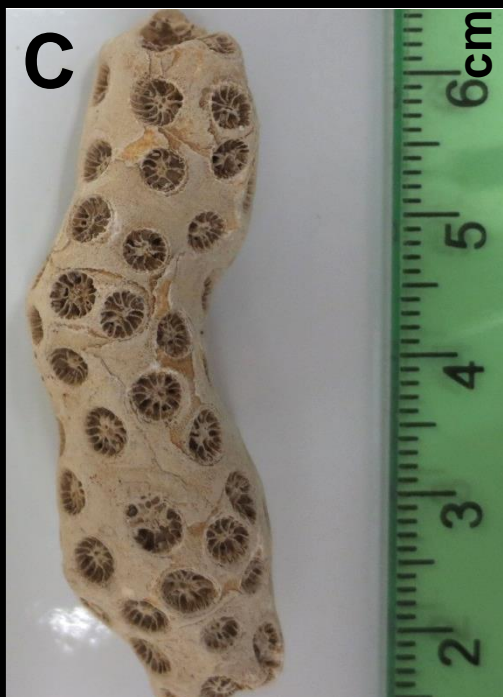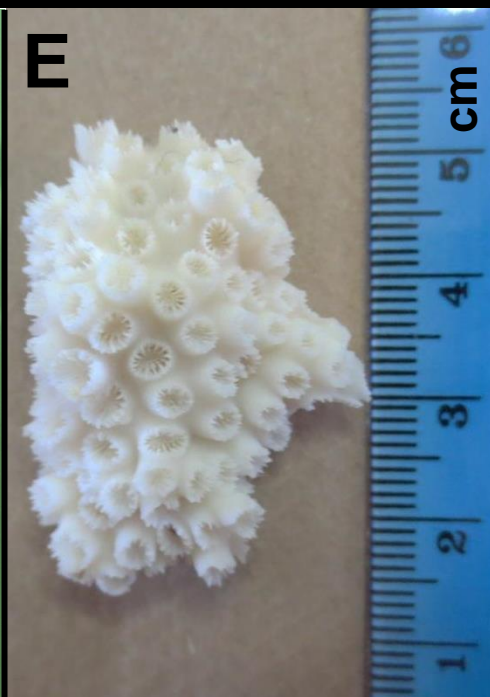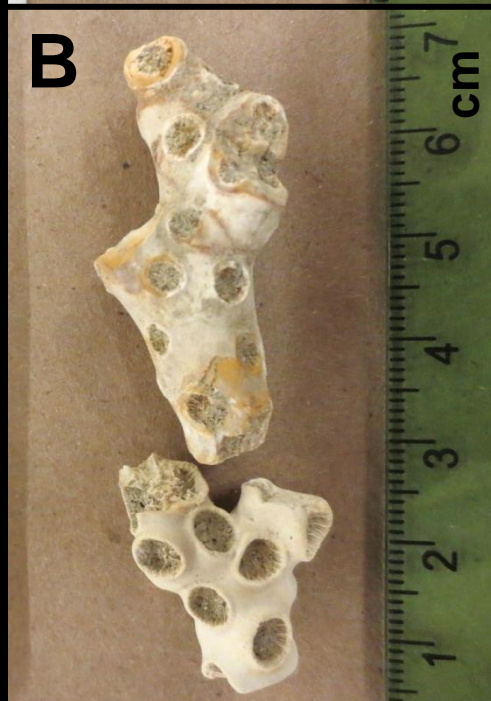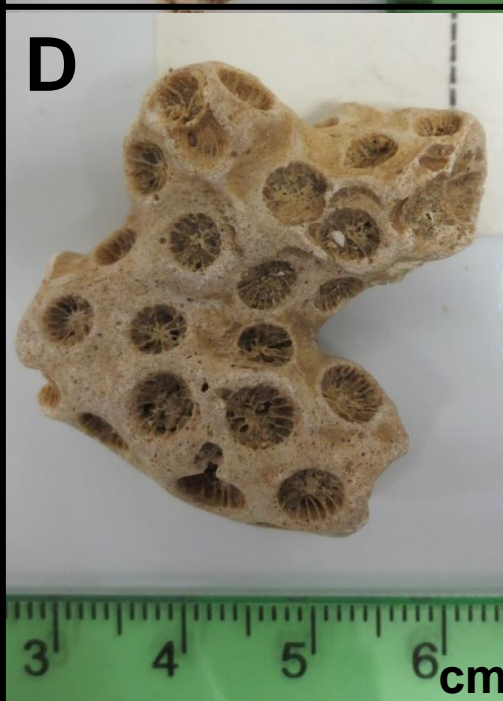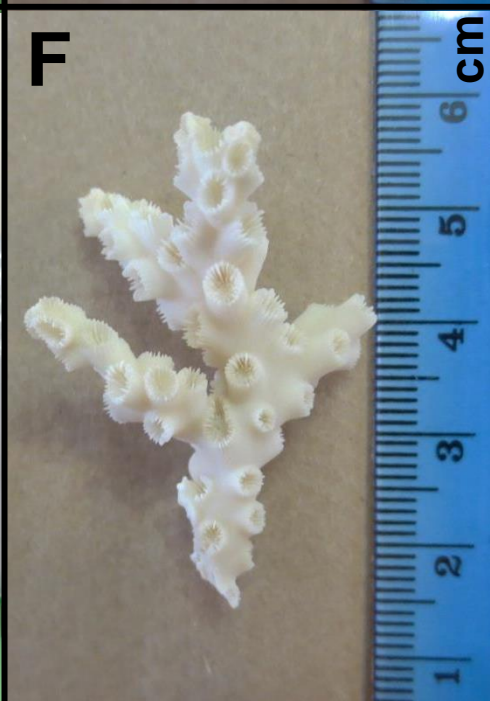

Supplement: Additional file 3: Figure S2. — Photographs of Oculina spp. specimens. A–D are Oculina spp. fossil specimens from the Smithsonian National Museum of Natural History. A and B are O. patagonica from South America (USNM 75199 and USNM 75205, respectively). C and D are O. crassoramosa from France (USNM I 80807). E is a skeletal specimen of extant O. patagonica from the eastern Mediterranean. F is a skeletal specimen of extant O. diffusa from Panama City, Florida (USA). [file 12862_2015_356_MOESM3_ESM.pdf]
