# Supplementary material for: The invasive coral Oculina patagonica has not been recently introduced to the Mediterranean from the western Atlantic
Source: BMC Evol Biol. 2015 May 5;15:79. doi: 10.1186/s12862-015-0356-7 (PMC4418043; doi:10.1186/s12862-015-0356-7)
Supplement: Additional file 2: Table S1. — Collection sites of all Oculina spp. samples used in this study. [file 12862_2015_356_MOESM2_ESM.docx]

**Table S2 - Nuclear markers used to genotype all *Oculina* spp. samples in this study**

| Marker | (putative gene)  Primer sequences | Length (bp) | # alleles | *S^a^* | *k^b^* | *π^c^* | *ϴ^d^* | rate*^e^* |
| --- | --- | --- | --- | --- | --- | --- | --- | --- |
| p14*^*^* | (Fatty acid elongase)  5′ TGTACCACTTGGGATGAACG 3′  5′ TCAAGCTTCCAGTCTTGTGAAA 3′ | 206 | 19 | 18 | 1.54 | 0.0075 | 0.0148 | 2.8 × 10^-7^ |
| p62*^*^* | (Elongation factor 1α)  5′ TGATTGTCCTCAACCATCCA 3′  5′ CTCCTGACAGACTTTCGATGG 3′ | 249 | 11 | 6 | 1.03 | 0.0042 | 0.0048 | 3.4 × 10^-7^ |
| p302*^*^* | (Tachylectin-2 motif)  5′ TTATACGGCGTCACAAACGA 3′  5′ TCGTCATCACCCTTTTATTCC 3′ | 226–229 | 20 | 28 | 2.17 | 0.0098 | 0.0228 | 3.2 × 10^-7^ |
| p243 | (Crystalline)  5′ TCCCCAGAATGTCAACAACA 3′  5′ ATTCYTTMCGAATGCTCTGC 3′ | 124 | 8 | 11 | 4.42 | 0.0357 | 0.0150 | 1.7 × 10^-7^ |
| p255 | (S-adenosylmethionine synthetase)  5′ GCCAGGTGGATTGCTAAGTC 3′  5′ CRTCTKTGTTTAAATAAAGCAAACATT 3′ | 197 | 18 | 18 | 3.91 | 0.0200 | 0.0155 | 2.7× 10^-7^ |

*^*^* Eytan et al. [23]

*^a^* segregating sites

*^b^* average number of nucleotide differences

*^c^* nucleotide diversity per site

*^d^* theta-W per site based on number of segregating sites

*^e^* substitution rate per locus per year used in IMa analyses
